# Ipsilateral Orolingual Angioedema Following Alteplase Administration for the Treatment of Suspected Acute Ischemic Stroke

**DOI:** 10.7759/cureus.38714

**Published:** 2023-05-08

**Authors:** Oluwaremilekun Tolu-Akinnawo, Oghanim I Ogwu, Ikenna Nnamani, Taiwo Talabi

**Affiliations:** 1 Internal Medicine, Meharry Medical College, Nashville, USA

**Keywords:** oroligual, ischemic stroke, ischemic cva, drug-induced angioedema, facial angioedema, oro-ligual, alteplase

## Abstract

Intravenous tissue plasminogen activator (tPA) remains the standard of treatment for patients presenting with acute ischemic stroke within the treatment window. In most patients, this often leads to an effective and life-prolonging intervention in the acute setting. This is, however, not without complications, which sometimes could be potentially fatal. Hemorrhagic complications, such as hemorrhagic conversion and bleeding, are the most discussed; however, facial angioedema has also been reported. We present a case of a 72-year-old African American male who developed right-sided ipsilateral orolingual angioedema 20 minutes after starting a tPA infusion. He was subsequently managed with antihistamine medications and steroids with interval resolution of symptoms. This case highlights the need for close monitoring while on tPA infusion, early detection, and management of potential facial angioedema complications. It also serves as a template for further studies focusing on preventative strategies for tPA-induced angioedema.

## Introduction

Stroke remains one of the leading causes of mortality and morbidity in both men and women worldwide [1]. However, more stroke cases are reported in women than men, which is attributable to the longer life expectancy for women [2-4]. About 795,000 Americans experience stroke-related events annually, with approximately 87% of cases being ischemic [4]. IV tissue plasminogen activator (tPA) was approved in 1996 for treating acute ischemic stroke and has since been the standard of care for patients who meet specific criteria [5]. Although hemorrhagic conversion has been well reported in the literature as one of the most typical and dangerous side effects of tPA administration, the occurrence of angioedema/ipsilateral orolingual angioedema is also now well documented at about 1.3-5% of stroke patients following tPA administration [6]. This case highlights another case of ipsilateral tongue swelling following alteplase administration, associated risk factors, its presentation, pathophysiology, acute management, and prognosis.

## Case presentation

The patient was a 72-year-old African American male with a past medical history significant for well-controlled hypertension, chronic alcohol abuse, osteoarthritis, and abdominal aortic aneurysm who was brought into the emergency department (ED) by the Emergency Medical Services (EMS) following a sudden fall from his motorized wheelchair one hour after consuming half a pint of vodka. He was found on the floor of his apartment by a concerned neighbor who had not seen him on the front porch and subsequently called EMS. On presentation to the ED, he denied loss of consciousness, head trauma, seizure-like activity, focal extremity weakness, palpitations, dizziness, vertigo, blurry vision/visual changes, and urinary incontinence/bowel incontinence. The review of systems was unremarkable except for slurred speech and fatigue. He was being planned for discharge from the ED when he suddenly developed a new-onset left forearm and leg weakness, which was maximal within several minutes of onset. He is a current smoker. He also drinks a half pint of vodka daily but denies currently using illicit substances (however admits to previous cocaine use). He had a known allergy to lisinopril. He had angioedema that occurred six years earlier and has since stopped taking the medication.

**Figure 1:**
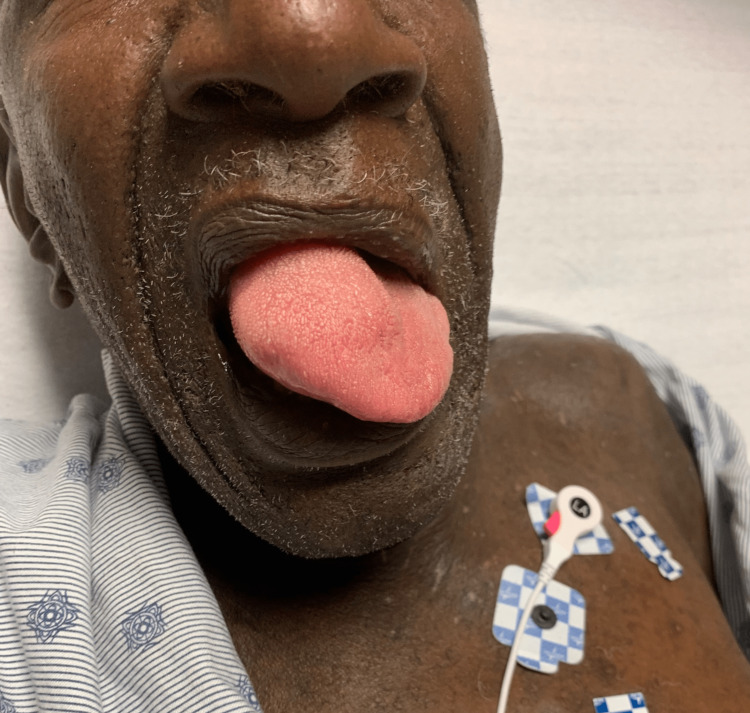
A picture of the patient showing significant right-sided tongue swelling following tissue plasminogen activator administration

Vital signs on presentation were unremarkable. Weight was 77.27 kg and body mass index (BMI) was 22.5 kg/m^2^ (reference: 18.5-24.9 kg/m^2^). Physical exam was significant for left forearm weakness of 3/5 muscle strength, left leg weakness of 3/5 muscle strength, and chronic left knee contracture; right-sided extremities had normal strength at 5/5. Deep tendon reflexes were diminished with no clonus. The random blood glucose obtained was 130 mg/dl (reference: 70-200 mg/dl). Due to suspicion of stroke (National Institutes of Health Stroke Scale (NIHSS) score was at least 9: blink eyes & squeeze hands +1 task, left arm motor drift +3, left leg motor drift +3, limb ataxia +1, dysarthria +1), a non-contrast CT of the head was done, which revealed no acute intracranial hemorrhage. CT angiography of the head showed significant arteriosclerotic calcification without evidence of hemodynamically significant stenosis and moderate stenosis at the C4-C5 level secondary to the posterior disc osteophyte complex. The neurology team evaluated the patient at the bedside. They recommended tPA administration of 70 mg, with 10% as a bolus and the remainder as an IV drip over the next hour, and volume expansion, with close monitoring in the intensive care unit (ICU). On arrival at the ICU, approximately 20 minutes after completing tPA administration (whole infusion), the patient complained of tongue swelling. On further evaluation, he was noted to have ipsilateral right-sided dorsal and ventral tongue swelling, which ends at the midline and without any respiratory compromise (Figure 1).

He immediately received IV diphenhydramine 50 mg, IV methylprednisolone 125 mg, and IV famotidine 20 mg with some interval symptom improvement. Anesthesiology was also emergently consulted; however, intubation was unnecessary. Tongue swelling remained stable without further progression over the next 48 hours. Transthoracic two-dimensional echocardiography (2D ECHO) with bubble study revealed an intact atrial septum without evidence of atrial septal defect (ASD) or patent foramen ovale (PFO). After 10 days of hospitalization, he was discharged on aspirin and high-intensity statin therapy with plans to continue rehabilitation with physical therapy (PT) and occupational therapy (OT) at a skilled nursing facility. Of note, no abnormal cardiac rhythm was noted throughout the course of hospitalization.

## Discussion

Alteplase (tPA) is a thrombolytic medication used to treat acute ischemic stroke, usually within 4.5 hours of symptoms onset. Alteplase emits its antifibrinolytic properties via the activation of plasminogen to form plasmin. The byproducts of these reactions lead to increased bradykinin production, complement cascade (C3a and C5a) activation, and increased histamine release. This has been explained as a possible mechanism responsible for angioedema in some patients following the administration of tPA. An increased risk of orolingual angioedema has also been reported in patients concurrently on angiotensin-converting enzyme inhibitors (ACEIs) due to increased circulating bradykinin in these patients [7]. Although our patient was not presently on ACEI, he had a previous orolingual angioedema reaction to ACEI in the past, leading to the discontinuation of the medication. This could explain an increased predisposition to tPA-induced angioedema in his case. The same study by Hill et al. also reported that seven out of the nine patients with tPA-induced angioedema had it on the contralateral side of the ischemic stroke, as noted in our patient [6]. The incidence rate of tPA-induced angioedema in a large study by Hill and Buchan was approximately 1.3%, which explains the rarity but the significance of the condition [7].

Due to the potentially life-threatening complications associated with tPA infusion, patient assessment should be done every 15 minutes during the infusion period for signs and symptoms indicative of clinical deterioration from hemorrhagic conversion/complications to facial angioedema [8]. Angioedema is a sudden but transient well-demarcated swelling involving the deeper layers of the skin, affecting the face, genitalia, and respiratory or intestinal linings [9]. Angioedema could also be secondary to hereditary deficiency of C1 esterase or allergic reaction to medications such as ACEIs, as in the case of our patient. Because the half-life of tPA is approximately seven minutes, angioedema reactions can still occur even after the infusion has stopped hence the need for continued monitoring. The initial evaluation of tPA-induced angioedema is airway evaluation and protection as needed due to the risk of extensive airway swelling that could occur with this condition. The tPA infusions need to be discontinued, and patients should be treated with antihistamine medications such as famotidine and diphenhydramine, alongside steroids, as used in the case of our patient [10]. Epinephrine and even bradykinin antagonists such as icatibant have also been previously used [5].

## Conclusions

This case highlights another incidence of tPA-induced orolingual angioedema and its clinical manifestation. Early detection provides key prognostic value, as early intervention can reduce the risk of respiratory compromise and even death. This highlights the need for close monitoring in patients undergoing tPA infusion. Further studies are needed to evaluate the genetic disposition to tPA-induced angioedema as well as racial predisposition, as this may guide preventative strategies.
